# Supplementary material for: Investigation and Source Apportionment of Air Pollutants in a Large Oceangoing Ship during Voyage
Source: Int J Environ Res Public Health. 2019 Jan 30;16(3):389. doi: 10.3390/ijerph16030389 (PMC6388280; doi:10.3390/ijerph16030389)
Supplement: Supplementary file 1 [file ijerph-16-00389-s001.pdf]

Supplementary

Table S1. Common factors and comprehensive scores of different sampling sites.

Sampling Site	Factor Score					Composite Scores
	VF1	VF2	VF3	VF4	VF5	
A	−0.75827	−0.3765	−0.05264	−0.35313	−0.37722	−0.43160
B	−0.65526	−0.27434	−0.13638	−0.32951	−0.35019	−0.37737
C	−0.45	−0.3219	0.09098	−0.23205	−0.27327	−0.26829
D	−0.19877	−0.30991	−0.43543	−0.20268	−0.04492	−0.22209
E	−0.53522	−0.29369	−0.15781	−0.27071	−0.20000	−0.32404
F	0.10631	−0.31627	0.28047	−0.83556	−0.19416	−0.09699
G	−0.48158	0.26521	−0.09933	−0.05197	−0.66087	−0.18028
H	0.37436	−1.6913	−0.96981	3.40615	0.35776	0.01739
I	−0.62232	−0.49449	−0.10455	−0.35531	−0.22058	−0.40437
J	3.33021	−1.29378	1.28444	−0.84664	−0.06165	1.00188
K	0.37893	0.58005	−0.53893	0.08314	−0.10291	0.20156
L	−0.37961	0.23048	−0.01741	−0.05673	−0.24457	−0.11240
M	−0.57514	−0.34032	0.12469	−0.57665	−0.4028	−0.35899
N	−0.50725	−0.12243	0.38961	−0.56038	3.79525	0.03582
O	0.51859	0.48207	−0.46046	−0.09662	−0.94245	0.16483
P	−0.26712	1.96151	3.0104	1.61012	−0.20722	0.88014
Q	−0.67081	−0.30276	−0.13022	−0.42966	−0.37517	−0.40086
R	1.39292	2.61836	−2.07762	0.09821	0.50498	0.87566
